# Influence of neurofeedback in improving the deaf students’ reading after cochlear implantation

**Published:** 2015

**Authors:** S Soltani Kouhbanani, R Khosrorad, M Hashemian, M Nasehnezhad

**Affiliations:** * Faculty of Education and Psychology, Ferdowsi University Of Mashhad, Mashhad, Iran,; ** Department of Education Development Center, Sabzevar University of Medical Sciences, Sabzevar, Iran,; *** Department of Health Education, School of Health, Sabzevar University of Medical Sciences, Sabzevar, Iran,; **** Health Education and Health Promotion, Mashhad University of Medical Sciences Mashhad, Iran

**Keywords:** deaf, cochlear implant, problems with reading, neurofeedback

## Abstract

The target of this investigation was to determine the effectiveness of neurofeedback in promoting reading in deaf kids after cochlear implantation. This research was an analytical example of 8 kids (5 sons and three girls) grown 8-14 years old by an IQ of eighty, based on Wechsler exam scheduled on the student’s list. Next recognizing the students according to specific standards, parents and their kid engaged in the research, which happened in Ava square in Ilam, and, afterward heard the stories about the way they could manage the trade themselves. No past of seizure disorders, epilepsy and brain trauma was recorded. No past of seizure disorders, epilepsy and brain trauma was recorded. The cochlear implant was done in 18 months and highest twenty-four months. The members were randomly split into a test collection and a check collection. The experimental group got neurofeedback therapy for twenty sessions of forty minutes any (3 sessions by week). The means used in this research were a demographic questionnaire (which covered data such as age, degree, and IQ), a reading disorder test, and the neurofeedback devices. The capability to read the neurofeedback collection recorded that the reading problems in the experimental group were lower. Also, the functional groups, relocation, replacement and reverse readings improved.

## Introduction

Regular hearing implies the capability to recognize the spoken words of others without any need to treatment aids or special procedures [**1**]. According to this definition, a person whose hearing has been damaged needs special procedures to understand the spoken words of others. The Education act has defined hearing disability as an audition impairment, which goes beyond so that the student will not be able to process the others’ verbal information without a hearing help. Hearing impairment has been reported among 28 million Americans, of whom, 1% has been seen with severe hearing impairment [**1**]. With regard to the investigations undergone by practitioners of the education center, in average, about 50% of the children require an education program and people with a hearing handicap require special aids. In this area, the main problem lies in how to train individuals who face limitation. Children with a hearing impairment cannot set relationships with others, thus the feelings such as isolation, failure and loneliness will dominate them, whereby this problem will complicate the issue of education for them [**2**]. Hearing loss is a reality throughout the world, which dates back to the literature on Exceptional Children. Factors, which have been considered in the area of deaf children, include discussion and exchange of ideas about sensorineural hearing loss, the oral methods, oral method with finger movements, oral (lip-reading) method, both sensory and multi-sensory methods, single sensory methods. Each of the proposed educational procedures for deaf children had consistent evidence; thus we could witness diversity in the educational styles in working with deaf children, where hopeful outcomes concerning efforts made to date could be observed [**3**]. About 30%-40% of deaf or hard-of-hearing babies have been seen with nerve growth status or mental retardation. According to the statistics research board, the most common statuses in hearing impairment include mental retardation, learning disabilities, and attention problems. Further, some disabilities might not emerge until childhood or adolescence, resulting in an increase of these figures. Despite the exposure with some similarities with mentally retarded children and children without mental retardation, in investigating general issues related to deafness in children, it could be observed that deaf children pass cognitive and linguistic stages similar to normal children, who repeated mistakes the same as mistakes by normal children at certain stages of evolution of language. Almost 30% of the kids with a hearing disorder, in addition to hearing loss, also have other disabilities including mental retardation, significant vision impairment, learning disabilities, and attention deficit disorder. Furthermore, emotional or behavioral problems, cerebral palsy, bone problems might also occur with lack of hearing. Looking into the previous experiences at the area of fostering children with newly diagnosed hearing impairment indicated that the early diagnosis of hearing disorder and use of intervention programs could be the best way to help for progress. To use the advantage of ductility of the central nervous organization, maximize treatment, and reduce functional defects, the intervention must be started, followed by the diagnosis. Children with an early hearing-loss detection and interruption related to the kids with late hearing-loss detection have better cognitive, social, and linguistic skills and all these factors help the children attend in normal conjunctive classes and open environments. In recent decades, intervention programs have been increasingly expanded to prevent behavioral disorders and healthy of mental problems of deaf children. Some of these programs could train the children with the required skills such as response inhibition, knowledge of impulsive behavior, emotion regulation. The committee of exceptional children and the council of deaf children have had some standards for teachers of deaf children for over one decade, in which all the needs required for deaf children were considered. Studies have shown that the kids who attended training courses for executive functions one or two years earlier, presented a huge progress in their social skills, skill of problem-solving and cognitive performance, such that all these progress related to executive functions [**4**].

Learning subjective subjects and theoretical contexts cannot be practicable, which is difficult for the character with a hearing disability and the reason for this is the impairment in speaking, thinking, and visualizing. Hence, it can be told that hearing impairment causes pseudo-academia and laziness. The studies on the academic performance of kids with hearing disability indicated loss academic performance of these children. In this regard, Peter Rimmer quoted from Saeedi in his studies, stating that he had perceived these children under their education level for 5 years. Using the Stanford Achievement Test (SAT.HT), which was standardized in 1972 on 6871 kids with hearing disability, Kretschmer found disorders of reading in these students. Reading is important education skills; it can be said that learning, knowledge, awareness, and advance in all the educational courses rely on the skill of reading. Reading, in all age groups, is one of the most influence methods of enriching language and expanding words, syntax, semantics, and language use. Reading disorder is one of the most common disorders in learning. Largest of the people with a reading disorder have been seen with loss progress in their brain cells and expanding problems in their reading derived from visual and auditory problems. This derives from problems in neurons, which have formed a network in the brain, deemed allocating for time changes. Reading, while one of the talents and abilities acquired in school, plays a major role in the individual growth of a character and his attendance in society. Reading is a cognitive and linguistic process, which has a familiar association with another linguistic process including speaking, writing, listening, which the child acquires. Notably, children with a hearing impairment face problems in their subjective activities such as processing, storage, and retrieval of data. A variety of studies have been conducted in the area of problems of reading comprehension among deaf children. In 1916, two English scholars [**5**] formulated a test to evaluate the reading skill, in which deaf students in the age group 14-16 years were asked to read some sentences and apply the instructions to the read the text, whereby the results indicated that the deaf students in the age group 14-16 years had the same function as the 7 years old students. In the procedure of knowing the reading comprehension input that was the text, finally, the semantic output was produced, whereby the meaning of the text was understood; thereby, the talent of reading without understanding the concept of terms was not accounted as a complete process to achieve the ultimate aim of reading, that was, the knowing of the meaning of the text. The linguistic processing must be fulfilled during special stages at different levels covering syntactic, phonological, and morphological words, thus the required ability for reading implies phonological awareness, reading comprehension and dominance on reading the words [**6**]. Due to deficits in working the memory, deaf students have a poorer comprehension and reading [**7**]. 

The outcomes of the research quoted by KakuJoybari [**8**] indicated that 0.95 of the deaf students who graduated from school, were deemed as 7 years old students regarding reading comprehension. 

Additional investigations determined that the deaf students face substantial problems in their life regarding verbal communication skills, such as understanding the meanings of words, speaking, reading comprehension, and writing [**8**]. Deaf education is the education of students with a variety of hearing levels, which dates back to a long time ago; teaching reading and writing is an unavoidable necessity to increase communication skills of hearing-impaired children. Reading is one of the cognitive skills that was developed as the result of the interaction between the nervous system and cultural experience [**9**]. Reading comprehension is a complicated procedure that requires coordination and combination of skills of word recognition and understanding the meanings [**10**]. Reading is one of the most important skills for children in learning courses. Proficiency in reading through speaking and listening or writing and reading comes to be realized by the use of signs and symptoms [**11**].

Studies in the meaning of deaf children’s reading comprehension have had disappointing results. It must be regarded that these results do not indicate an intellectual disability of deaf children, but the weakness in the reading comprehension in this group derives from the factors with cognitive nature. Reading comprehension is both a cognitive and linguistic process, which has a close relation with the another linguistic processes including speaking, writing, listening, which the child acquires. Notably, the children with a hearing impairment face problems in their subjective activities such as processing, storage, and retrieval of data. Since reading comprehension and learning reading skill require the acquisition of visual recognition of linguistic elements including letters, words and sentences, thus recognizing strengths and weaknesses in both cognitive and linguistic areas, it seems that essential brain waves depending on the frequency are classified into four groups: Delta (1 to 3 Hz), theta (4 to 7 Hz), alpha (8 to 13 Hz) and beta (14 to 30 Hz). We can witness alpha activity when a person is relaxed, but alert. Yet, when a person involves in a cognitive activity or problem resolving, we can witness beta waves. Delta waves are recognized when people are in long sleep or in a coma, and theta waves are seen when the person is in light sleep [**12**]. Neurofeedback is a treatment model for changing or modifying cognitive, emotional, and physiological processes in patients. The results of studies indicated that neurofeedback fosters the brain for activity or proper pattern during various sessions. However, a variety of studies on treatments for learning disabilities concerning neurofeedback have been conducted; in a study it was indicated that children with learning difficulties are different from other children in terms of EEG indicators. One of the methods for the normalization of the brainwave of children with learning disabled (LD) can use Valproate Sodium. Neurofeedback therapy is another technique that normalizes brainwaves. Neurofeedback regards to the method of factor conditioning in which the individuals learn to change the electrical activity of their brain [**13**]. Neurofeedback aims to treat EEG abnormalities, whereby improvement of cognitive or behavioral performances will result. Neurofeedback is inhibited as a form of conditioning the electrical action of the brain. It is believed that neurofeedback recalls growth and brings about alterations in brain cell surfaces, which supports the brain function and the cognitive/ behavioral performance [**12**].

The sensorimotor cortex helps the cerebral cortex in the contemporary production of physical and cognitive responsibilities, and this comes out of executive performances. It can be understood why early pioneers in the neurofeedback area have started the education process during the sensorimotor cortex. 

Further, Ratey [**14**] mentioned that brain circuits that are used to regulate a subjective practice are those used to regulate a physical practice, i.e. The sensorimotor cortex works out in conducting physical and mental processes, and this cortex is more likely used for sensorimotor functions. Hence, the clients who have difficulties in understanding their cognitive tasks can use the neurofeedback education in their treatment process. Neurofeedback education using the systems which deal with emotion, feeling, attention and working memory, develops the energy source, movement, reasoning and thinking. Regarding the SMR area, in another explanation for finding of this research, it can be told that the activation of neuronal circuits involved in cognitive processes comes to be realized. Previous investigations have explained that the working memory depends on a neuronal circuit, which is acquired from the interaction between the attention control system in the prefrontal cortex and the sensory information storage in the dorsal prefrontal association area. In current research, a part of. In current research, a part of the protocol was applied for the destruction of theta waves, and the related works showed that theta relates to poor performance, and the outcomes showed that the suppression of theta waves caused a better cognitive performance. In other words, neurofeedback education has positive effects on the individuals’ subjective performance and cognitive processing, confirming the conclusions of this research. 

Another explanation for the effect of neurofeedback thalamus, regarding the early changes in the activity, more likely occurring via neurofeedback, mentioned that these changes might modify EEG through thalamocortical circuits. Hence, changes in the EEG were deemed as the result of complex reorganization of neuronal activity. A variety of studies have been conducted in the area of effectiveness of neurofeedback in developing the students’ reading difficulties, whereby significant results have been obtained [**15**,**16**]. To date, this method has not been used to improve the comprehension in deaf children. In this research, the writers tried to get an approach to increase the reading comprehension of deaf children so that to help improving the educational, communicative, and social abilities of deaf children, besides other educational approaches of deaf children. 

**Research method**

A semi-experimental study was applied in current research. After the cochlear implantation, the deaf students who have accomplished their auditory training therapies or treatments such as auditory and linguistic proceeding in the Ava Center in Ilam were selected as the sample group. The inclusion criteria include DSM-IV diagnosis of the reading disorder and inclusion tests to measure the reading disorder. 8 students (five sons and three daughters) in the age group of 8-14 years old with an IQ of eighty regarding the Wechsler test, were involved in the research. Next knowing the students with the inclusion criteria, their fathers and mothers played in the Ava Center in Ilam and after hearing the explanations, they tried to manage the trade themselves. The addition measures involved the following: not having any medical history of diseases of epilepsy, seizures, and brain damage. About 18-24 months must have given because the cochlear implantation, and the age of participants had to be within eight and fourteen aged. At first, members elected in the in the demographic questionnaire with the aid of their teachers and parents, and then the reading test took place, the researcher taking them in a calm room. The voice recorder was applied to register contents, and the timer was applied to register the reading time in the tasks relating to speed of calling and reading. Then, the members were split into 2 test and control groups; the test group got neurofeedback in 20 sessions during 40 minutes, and the control group just stared at purposeless images on the screen of a computer. Finally, the groups played in a post-exam of reading disorder, and then the results were considered after one month.

**The tools applied in this research**

**Demographic questionnaire (information on age, education level, and IQ)**

**Reading disorder test**

The reading disturbance exam developed and normalized by Nesfat et al. [**17**] was used to diagnose kids with reading problems. According to this test, three clinical characteristics including mistakes in reading, speed of reading and reading knowledge of students were measured. According to this test, a score was given to each participant per any error, and the sum of scores of the member was recognized as his total errors; further, a score was awarded to each participant per any response to the reading knowledge problems. Finally, the time spent by the participant from the start to the end of reading the text, was regarded as the index of speed of reading; further, coefficients of reading validity of 0.56, 0.61 and 0.68 at third, fourth and fifth grades were obtained [**17**].

Neurofeedback device: the neurofeedback device was used for two purposes: 1-to register brain waves, 2- to present feedback. The device used in this research included five channels named ProComp 5.5 made in Canada, with its sensitivity of sampling equal to 256 Hz. In this research, two treatment protocols were applied for the target of treatment by using the neurofeedback therapy in kids with reading difficulties. 

**Neurofeedback therapy**

Neurofeedback therapy is the initial treatment protocol applied for the suppression of alpha/ theta in the Cz, aiming at increasing alpha and reducing theta. 

The second protocol: the SMR protocol was implemented in C3, C4 areas. In this protocol, SMR (subset of beta) was strengthened for 12 to 15 Hz, and theta was suppressed for 4 to 7 Hz. These two protocols were repeated during all 20 education sessions. Since session seven, any student seen with progress, received a CD box. They also received a CD in the thirteenth and nineteenth sessions. The rational reason for the treatment used in this research relied on the factors below: in comparing EEG in normal and LD children, the highest amount of EEG frequency was in LD children by increasing the theta activity [**18**-**20**]. In the second protocol, theta (407) and hyper-beta (22-30), suppression of SMR (subset of beta) in encoding dynamiv and cognitive responsibilities helped the cerebral cortex, adding that the encoding of dynamic and cognitive responsibilities helped the cerebral cortex in brain circuits. Brain circuits which were used to regulate a subjective practice, were those used to regulate a physical practice. Hence, the clients who had difficulties in understanding the logical sequence of cognitive tasks could use the neurofeedback therapy in their sensorimotor cortex of the right hemisphere (C4) to recall their emotions and feelings. Education in midpoint or Cz facilitated a mixed response. In other words, increasing the activity at the central cortex was followed by increasing the activity in skills of both hemispheres as a precondition for a prosperous understanding and attainment of the reading skills. Neurofeedback sessions were organized until the twelfth session during three sessions in a week, and sessions of 12 to 19 were organized during two sessions in a week. Treatment processes: the participant was sat on a chair in front of a computer screen in a room in a total silence; he prepared tulip ear-tips and areas C4, C3, Cz by using alcohol and gel, for which Alpha-theta protocol was performed. To this, the active electrode was transferred to point Cz. Feedback of alpha-theta protocol was in the form of voice (sound of waves in ocean - River), that the participant used to hark to the sound with his eyes closed, and if he fell asleep, the device had to be alerted. In this protocol, the person found the ability to establish a balance and coordination between alpha and theta, where this protocol was performed in any session during 20 minutes. The next 20 minutes were considered for training protocol SMR, where theta waves of 4-7 Hz and beta waves of 22-30 Hz were suppressed, and SMR waves were strengthened. Finally, the threshold was written down for the next session.

**Findings**

**Table 1:** The characteristics of the experimental and control groups

	The experimental group (n = 4)		The control group (n = 4)		
	Mean	Standard deviation	Mean	Standard deviation	Significant level
Age	11.23	11.23	11.9	14.12	0
IQ	94.8	8.48	89.6	7.6	0

**Table 1** shows the coordinated characteristics of students in 2 groups. The T test shows no significant discrepancies among the trial and control groups.

**Table 2:** The outcomes of London Tower test in rein and experimental groups

	The experimental group				The control group				
Elimination	pre	post	deviation	errors	pre	post	deviation	errors	Significant level
add	2/ 53	0/ 98	2/ 0	1/ 08	2/ 09	1/ 99	0/ 48	0/ 54	0/ 05
relocation	4/ 82	3/ 39	1/ 11	1/ 43	4/ 64	4/ 14	0/ 78	0/ 90	0/ 05
replacement	10/ 53	5/ 4	0/ 92	1/ 33	7/ 65	10/ 15	9/ 48	1/ 52	0/ 05
mirror reading	12/ 34	7/ 6	1/ 35	1/ 19	12/ 71	11/ 11	1/ 17	0/ 65	0/ 05

**Table 2** represented the results of neurofeedback therapy on the reading comprehension of two test and handle groups in pre-exam and post-exam. The outcomes showed that the reading knowledge of the test group went beyond the control group, where that difference lied on adding. The results indicated that the displacement in the test group went beyond the rein collection, and that variation was important in adding. Further, the displacement in the test group went beyond the control group, and that difference was significant. The reading inversion in the test group went beyond the control group, and that difference was significant.

## Discussion and conclusion 

Besides all the studies on exceptional individuals, on linguistic, cognitive and verbal abilities of deaf individuals and their comparison with normal individuals to examine the differences, have been more likely drawn into attention in scientific communities. bout the ability of deaf individuals concerning the achievement of life skills, attention to training these individuals is important. Deaf children often cannot foster the courses and concepts relating to language in them. If hearing impairment occurs at the early childhood, the deaf child might find some of the various sorts of learning impossible. As the early experiences of the kids are often visual and tactile, their first dialogues are generally through sign language and gestures. The children who have hearing difficulties, learn in their early childhood how to talk with so much difficulty, because proper dialogue relies on copying from others’ speech. Hearing impairment affects the formation of a concept. Progress in reading has a slow trend in person including a hearing disability [**8**], who believes that reading comprehension in deaf children is poorer compared to normal children. 

The studies in the two recent decades have given use of absolute responses, the responses that have confused the teachers and have obliged them to have a revision on their methods about fostering deaf children. By studying brain injuries and their complications on reading, some researchers called important brain activities involved in brain injuries. A special electroencephalogram (EEG) pattern has been observed in children detected with dyslexia [**19**]. It was revealed that there is a meaningful distinction with common children and children affected by a reading disorder concerning EEG waves. Neurofeedback seeks to train the individuals to normalize their brain waves reaction to the stimulants. Neurofeedback can be applied to stimulate or regulate the exercise of the brain. Neurofeedback is also used for normal individuals’ neurofeedback causes increasing the capacity of working memory and attention to education performance, and besides, neurofeedback had significant results in LD treatment. The therapists enabled to indicate that training neurofeedback could cause an enhancement of the cognitive functions. These results are relevant and similar to the conclusions of the research by Vernon et al [**5**]. A section of the protocol applied in this research was regarded as the increase of Cz. To define this finding, it can be said that neurofeedback education affects three sensorimotor cortexes. Therefore, it can be assumed that neurofeedback facilitates information processing because SMR reduces the voluntary direction of the motor system interference in Cognitive Information Processing.
